# White-tailed deer S96 prion protein does not support stable in vitro propagation of most common CWD strains

**DOI:** 10.1038/s41598-021-90606-8

**Published:** 2021-05-27

**Authors:** Alicia Otero, Camilo Duque Velásquez, Judd Aiken, Debbie McKenzie

**Affiliations:** 1 Department of Biological Sciences, University of Alberta, Edmonton, AB Canada; 2 Centre for Prions and Protein Folding Diseases, University of Alberta, Edmonton, AB Canada; 3 Department of Agricultural, Food and Nutritional Sciences, University of Alberta, Edmonton, AB Canada

**Keywords:** Ecological epidemiology, Emergence

## Abstract

PrP^C^ variation at residue 96 (G/S) plays an important role in the epidemiology of chronic wasting disease (CWD) in exposed white-tailed deer populations. In vivo studies have demonstrated the protective effect of serine at codon 96, which hinders the propagation of common CWD strains when expressed in homozygosis and increases the survival period of S96/wt heterozygous deer after challenge with CWD. Previous in vitro studies of the transmission barrier suggested that following a single amplification step, wt and S96 PrP^C^ were equally susceptible to misfolding when seeded with various CWD prions. When we performed serial prion amplification in vitro using S96-PrP^C^, we observed a reduction in the efficiency of propagation with the Wisc-1 or CWD2 strains, suggesting these strains cannot stably template their conformations on this PrP^C^ once the primary sequence has changed after the first round of replication. Our data shows the S96-PrP^C^ polymorphism is detrimental to prion conversion of some CWD strains. These data suggests that deer homozygous for S96-PrP^C^ may not sustain prion transmission as compared to a deer expressing G96-PrP^C^.

## Introduction

Chronic wasting disease (CWD) is a prion disease affecting multiple species of cervids in North America, South Korea, and Scandinavia. Prion diseases, which also include scrapie in sheep and goats, bovine spongiform encephalopathy (BSE) in cattle and Creutzfeldt-Jakob disease (CJD) in humans, are fatal diseases produced by the aberrant misfolding of the cellular prion protein (PrP^C^) into an infectious isoform (PrP^CWD^) which accumulates in the brain causing neurodegeneration^1^. PrP^CWD^ is resistant to protease K (PK) digestion producing a resistant core referred to as PrP^Res^ [reviewed by^2^]. PrP^C^ is encoded in all mammalian species by the *PRNP* gene and is expressed in numerous organs with the highest levels found in the central nervous system (CNS)^3^. Unlike most prion diseases, CWD is highly contagious among farmed and wild animals, spreading through animal-to-animal interactions and exposure to contaminated environments^4–7^.

The primary sequence of the prion protein is one of the main determinants of the susceptibility to prion diseases in numerous species, including humans, mice, sheep, goats and cervids. In sheep, the susceptibility to classical scrapie is largely regulated by amino acid variations (polymorphisms) at positions 136, 154 and 171 of the prion protein. The expression of alanine (A), arginine (R), arginine (R) at these positions provides great resistance to the disease^8–11^. Accordingly, breeding programs aimed at eliminating animals expressing susceptible alleles and increasing the frequency of animals expressing the A136/R154/R171 haplotype have been implemented in sheep herds from countries where scrapie is endemic. This selective breeding for resistance has resulted in a significant decrease in scrapie prevalence, reducing it to almost zero in some cases^12–14^. A similar approach has been proposed to control and/or eradicate classical scrapie in goat populations. This breeding program, although not yet implemented, is based on the selection of animals expressing the K222 *PRNP* allele, which provides a level of resistance to scrapie equivalent to ARR in sheep^15–18^.

In cervids, there is also a close relationship between polymorphisms of the *PRNP* gene and CWD infection status. Supplementary Figure 1 shows sequence alignment of PrP^C^ polymorphisms in cervids commonly affected by CWD. The most common *PRNP* allele found in white-tailed deer populations affected by CWD encodes PrP^C^ molecules with glutamine (Q), glycine (G), alanine (A) and glutamine (Q) at positions 95, 96, 116 and 226, respectively, and is designated as the wild-type (wt)^19–25^. Epidemiological studies in CWD endemic areas have shown that white-tailed deer expressing certain amino acid variations such as S96, H95, and G116 are underrepresented among CWD positive animals suggesting a protective effect against the disease^26–30^. The protective effect of S96 and H95 alleles was further demonstrated by experimental oral infection in white-tailed deer expressing these amino acid substitutions^19^. Among the alleles of the *PRNP* gene associated with a lower CWD incidence and extended preclinical phase, S96 has the highest allelic frequency (~ 25%) after the wt allele in several white-tailed deer populations from the United States and Canada^26,27,31^. Subsequent independent transmission and epidemiological studies have demonstrated that deer homozygous and heterozygous for S96-PrP^C^ are, compared to wt/wt deer, less susceptible to CWD infection, present prolonged survival times, show delayed prion accumulation and are generally at a significantly earlier stage of disease when deer herds are depopulated^23,31–33^.

Intracerebral challenge of mice expressing S96-PrP^C^ with numerous CWD isolates has resulted in no disease^21,22^ or incomplete attack rates^34^, corroborating the impact of this polymorphism on CWD infection. The same S96 (tg60) mouse line is, however, susceptible to the challenge with the CWD strain H95^+^, which originated from the passage of Wisc-1 CWD prions from wt/wt deer into deer expressing H95-PrP^C^^25^. Natural infection of white-tailed deer expressing G116-PrP^C^ also resulted in different strains^35^. These polymorphisms have been reported at relatively lower frequencies in wild and captive deer populations^27,28,30,31^. By contrast, the S96-PrP^C^ allele could become dominant in white-tailed deer populations exposed to CWD within a short time frame^36^.

Here, we compared the efficiency with which different CWD strains induce and maintain the in vitro misfolding of white-tailed deer G96- and S96-PrP^C^ molecules. Previously, cell-free conversion assays showed that S96-PrP^C^ can be converted into PrP^CWD^ as efficiently as wt-PrP^C^ when incubated with CWD seeds from different cervid species^37^. Similarly, we observed that deer wt-PrP^C^ and S96-PrP^C^ are converted by different CWD strains with similar efficiencies after one round of PMCA. However, while all these CWD strains self-propagate efficiently in the wt-PrP^C^, they could not sustain serial propagation in the S96-PrP^C^ substrate. These results suggest that the most common CWD strains cannot stably template their conformation on S96-PrP^C^, leading to the extinction of prion conversion after a few rounds of in vitro amplification in this substrate. These results reinforce the importance of the S96 allele as a candidate for selective breeding programs aimed at controlling and eradicating CWD in wild and farmed white-tailed deer populations.

## Results

### CWD strains replicate with similar efficiencies in a single round of PMCA with wild type and S96-PrP^C^

Traditionally, the method for quantitatively estimating the infectivity of a prion isolate is end-point dilution titration in animals, generally transgenic mice expressing the prion protein of interest. This method is, however, time-consuming, and expensive. Therefore, numerous studies have relied on protein misfolding cyclic amplification (PMCA) to estimate prion infectivity in vitro. PMCA allows precise titration of prion agents in a few days significantly reducing the number of animals required^38–41^.

We used PMCA to estimate the infectivity of different CWD strains by determining their limiting dilution (i.e., the highest dilution capable of producing detectable prion amplification) in brain substrates prepared from tg33 and tg60 mice. Tg33 mice express deer wt-PrP^C^ (i.e., G96-PrP^C^) at levels ~ onefold compared to deer brain, whereas the PrP^C^ expression level in tg60 mouse brain is about 30% lower than in tg33^21,22^. Therefore, to obtain more comparable results, we adjusted the PrP^C^ content of the tg33 substrates by diluting the tg33 brain homogenate in brain homogenate from *Prnp*^*0/0*^ mice.

To compare the propagation efficiency of different CWD strains in wt and S96-PrP^C^, 10% (wt/vol) brain homogenates from cervids infected with Wisc-1, H95^+^^25^ or CWD2^42^ strains, and a brain homogenate from a mule deer naturally infected with CWD (MD inoculum) were used to prepare serial tenfold dilutions.

The Wisc-1 and H95^+^ strains were obtained from two experimentally inoculated white-tailed deer, one wt (Q95G96) homozygous and another expressing different H95/S96 PrP^C^ molecules, respectively^19,25^. The specific PrP^CWD^ conformations of these two strains have been linked to differences in host range and reproducible neuropathological, biochemical and biophysical signatures upon serial transmission into experimental hosts^24,25,43^. The CWD2 strain was obtained from orally inoculated elk of the MM132 genotype^44^ and it was classified as a different strain based on incubation periods and histopathological features in transgenic mice^42^. The MD inoculum was obtained from a naturally infected free ranging wt/wt mule deer from Alberta, Canada and was included for comparison with the Wisc-1 given the similarity in PrP^C^ primary sequence and PrP^Res^. Bioassay characterization of this MD isolate is ongoing in tg33 and tg60 mice.

**Figure 1:**
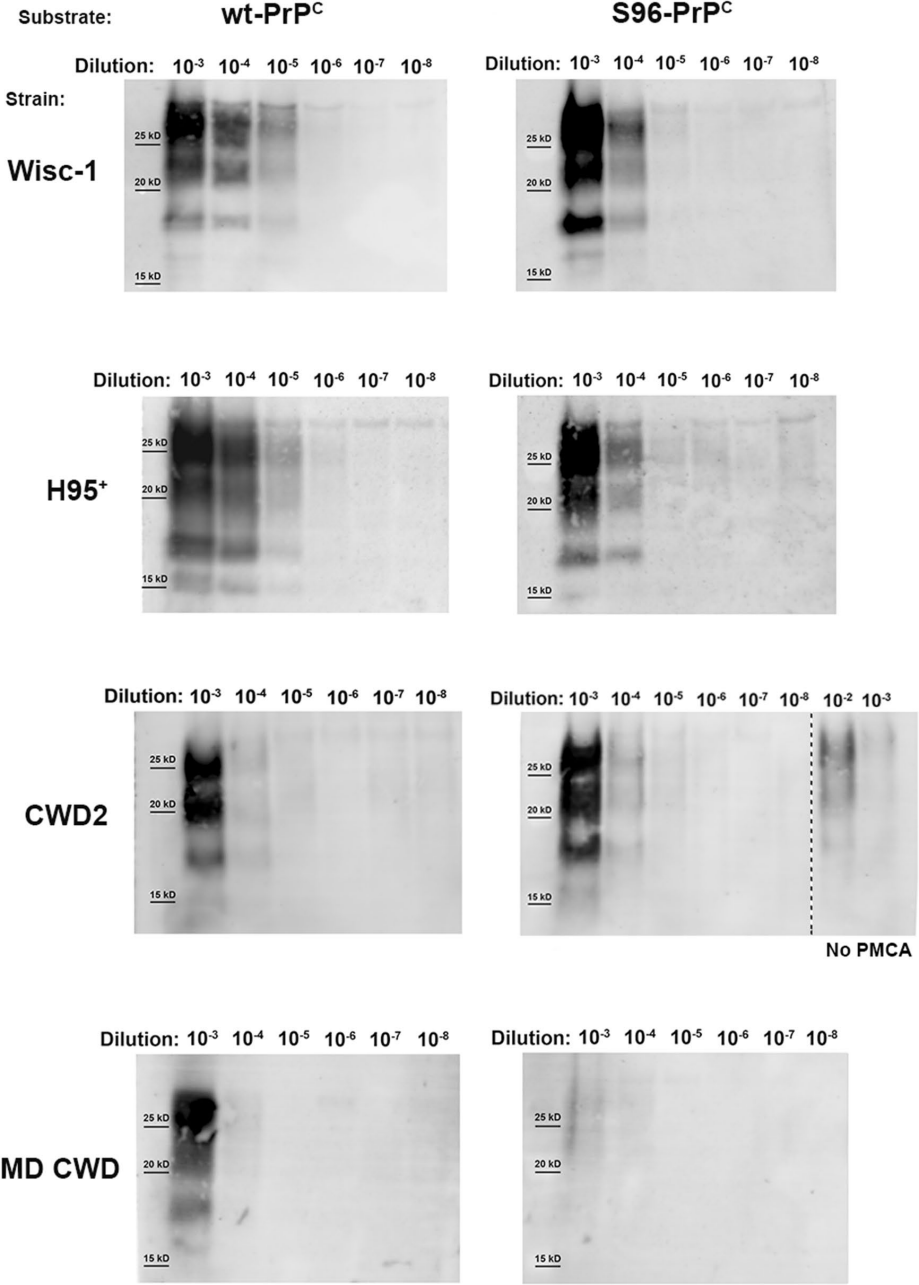
PMCA amplification efficiency of different CWD isolates in wild-type (wt) and S96-PrP^C^ substrates. Serial dilutions (10^−3^ to 10^−8^) of white-tailed deer CWD strains Wisc1 and H95^+^, CWD2 strain from elk and a CWD isolate from a naturally infected mule deer (MD) were subjected to PMCA in wt-PrP^C^ or S96-PrP^C^ brain homogenate substrates (tg33 and tg60 mouse brain homogenates, respectively). PMCA products were digested with PK (50 µg/ml) and analyzed by western blot. Wisc-1 and H95^+^ strains propagated more efficiently in the wt-PrP^C^ substrate. CWD2 strain propagated with the same efficiency in both wt and S96-PrP^C^substrates. A 10^-2^ and a 10^-3^ dilution of the CWD2 strain in brain homogenate, not subjected to PMCA, is shown as a control of PMCA amplification. MD CWD only propagated in the substrate expressing wt-PrP^C^. mAb Sha31 1:10,000.

Aliquots from each dilution were used to seed the wt or S96 substrates. After a single round of 24 h of PMCA, reactions were analyzed for PrP^CWD^ amplification. Following one round of PMCA, both Wisc-1 and H95^+^ prions were detectable up to the 10^−5^ dilution in the wt substrate and up to the 10^−4^ dilution in the S96 substrate (Fig. 1). Elk CWD2 prions propagated with the same efficiency (10^−4^ dilution) in both substrates. MD CWD only propagated in the wt substrate and only up to the 10^−3^ dilution, suggesting that white-tailed deer prions propagate more efficiently in both substrates. Alternatively, the Wisc-1 prions have higher seeding activity because they were obtained from a terminally ill experimental animal while the MD isolate was from a hunter-harvested deer submitted for CWD surveillance. Interestingly, when compared by western blot, the MD isolate had approximately twice the PrP^Res^ content compared to the Wisc-1 (not shown). Our results suggest that different CWD isolates propagate with similar efficiencies in both wt and S96 substrates after one round of in vitro prion amplification.

### Wisc-1 and elk CWD2 attenuation through serial PMCA on S96-PrP^C^

Serial PMCA is a technique that enables replication of the abnormal, pathological prion protein (PrP^CWD^ in CWD) in vitro. After one round of PMCA, an aliquot of the amplified sample is diluted tenfold into fresh brain homogenate substrate and subjected to another round of PMCA. This process can be repeated, obtaining newly converted PrP^CWD^ which is able to induce PrP conversion with similar efficiency as brain-derived PrP^CWD^^45^.

**Figure 2:**
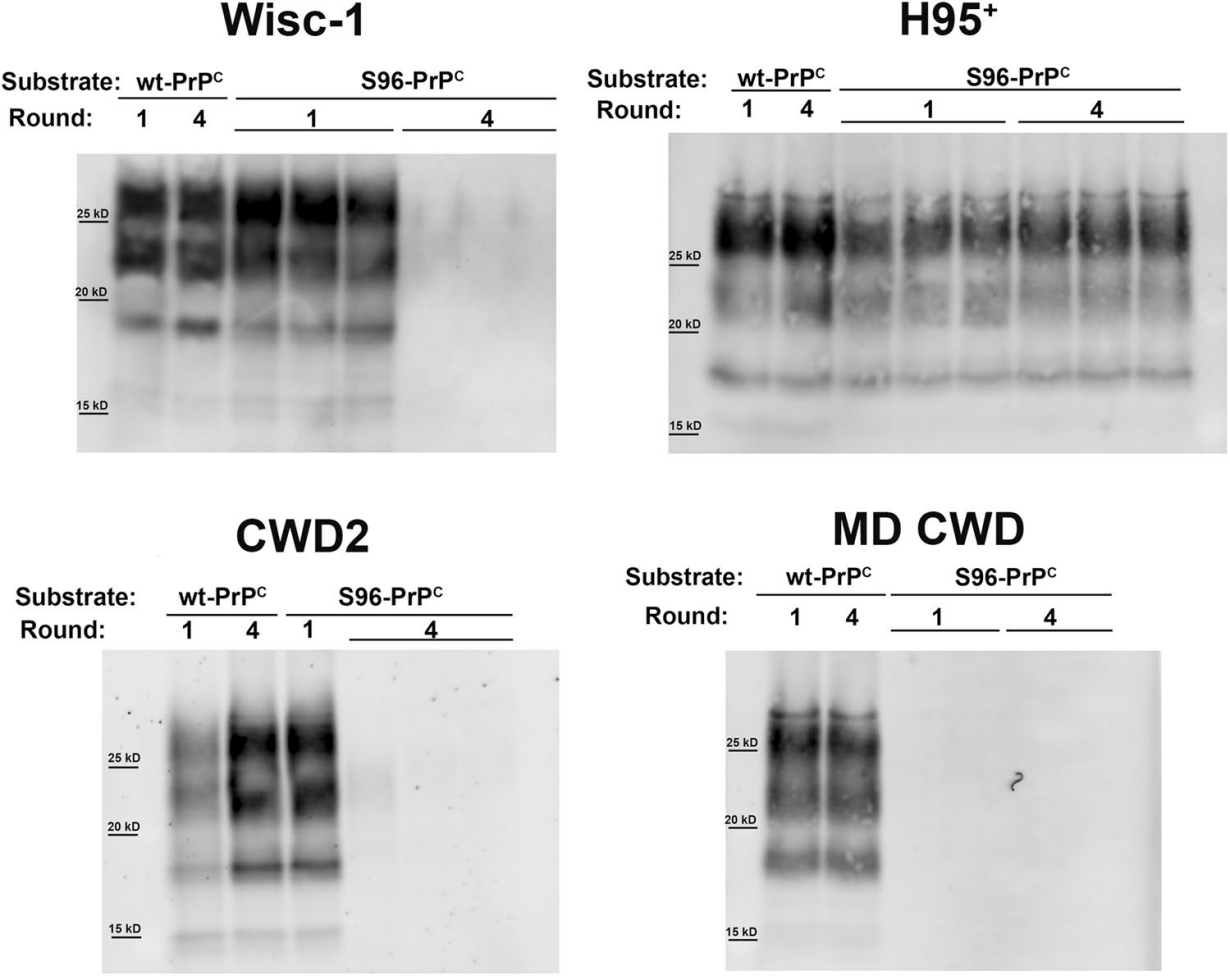
Serial PMCA amplification of Wisc-1, H95^+^, CWD2 and MD CWD prions in wt and S96 PrP^C^ substrates. 10^−3^ dilutions of the CWD brain homogenates from infected cervids were used to seed wt-PrP^C^ substrate (tg33 mouse brain homogenate) or S96-PrP^C^ brain substrate (tg60 mouse brain homogenate) and subjected to 4 serial rounds of PMCA. Amplification products were digested with PK (50 µg/ml) and analyzed by western blot. Although all CWD strains were serially propagated in the wt-PrP^C^ substrate, only the H95^+^ strain maintained its amplification efficiency in the S96-PrP^C^ brain substrate through the serial rounds of PMCA. Wisc-1 and CWD2 strains lost their ability to misfold the S96-PrP^C^ after serial rounds of PMCA. MD CWD isolate was not able to propagate in this substrate. mAb Sha31 1:10,000.

Wisc-1, H95^+^, CWD2 and MD CWD prions were subjected to 4 rounds of PMCA, in triplicate, in wt or S96-PrP^C^ brain substrates. Analysis of the last PMCA round demonstrated that all CWD isolates propagated efficiently in the wt-PrP^C^ substrate, showing abundant PrP^CWD^ that was biochemically indistinguishable from the original brain-derived seed. Surprisingly, no PrP^CWD^ was detected in the final round of Wisc-1 and CWD2 amplification in the S96-PrP^C^ substrate. These results were unexpected, considering that these isolates propagated with similar efficiencies in wt and S96 substrates in the first round of PMCA. The MD CWD isolate was not able to propagate in the S96 substrate. Seeding of the S96-PrP^C^ with the H95^+^ strain, however, resulted in successful serial prion propagation (Fig. 2).

**Figure 3:**
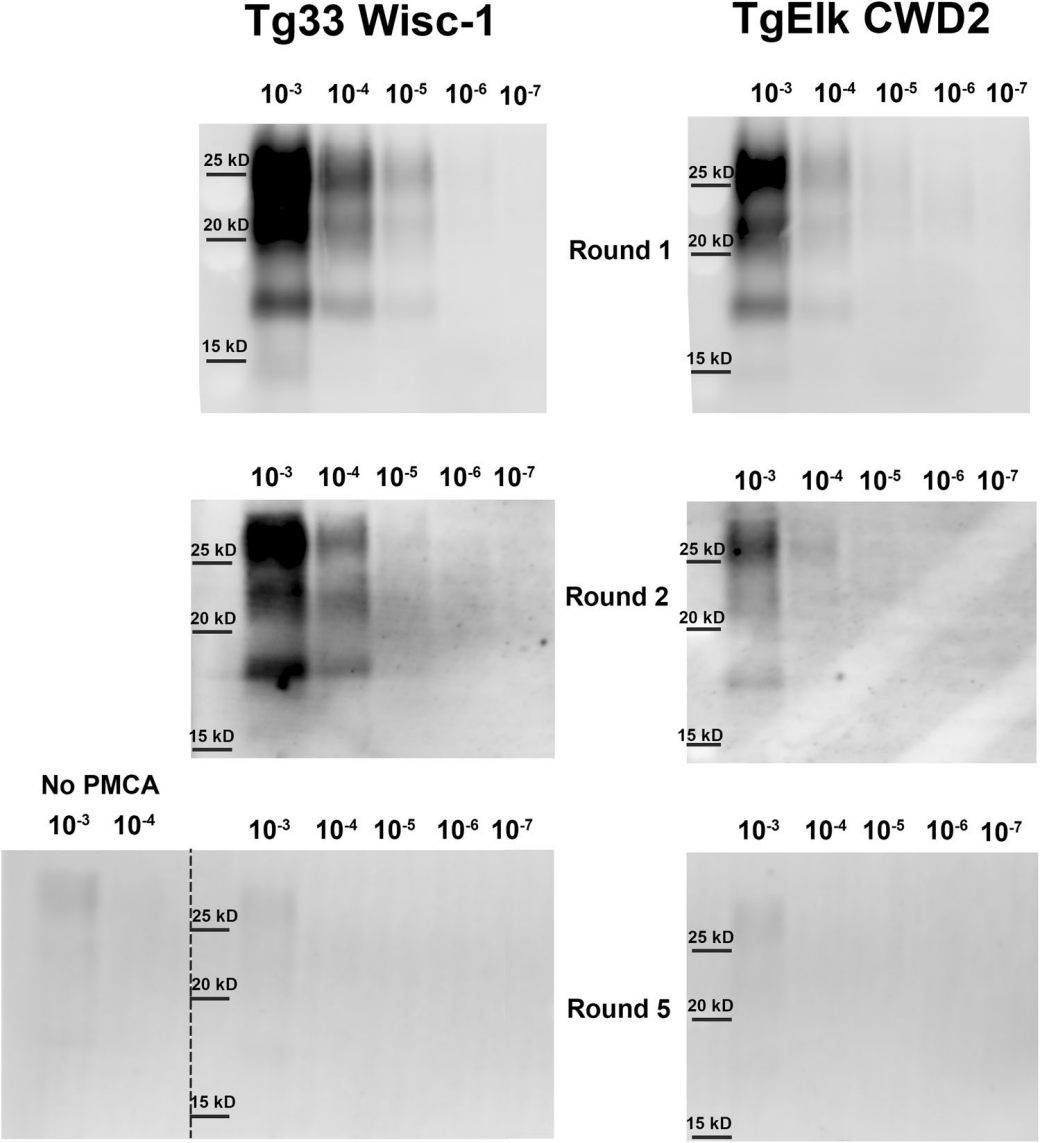
Serial PMCA amplification of tg33 Wisc-1 and tgElk CWD2 prions in S96-PrP^C^ substrate. Serial dilutions (10^−3^ to 10^−7^) of brain homogenate from a tg33 mouse (expressing white-tailed deer wild-type PrP) infected with Wisc-1 CWD strain and a tgElk mouse (expressing elk PrP) infected with the CWD2 strain were serially propagated in the S96-PrP^C^ brain substrate (tg60 mouse brain homogenate) for 5 rounds of PMCA. PMCA products were digested with PK (50 µg/ml) and analyzed by western blotting. Amplification efficiency of both strains was reduced in one log after two rounds of PMCA. After 5 rounds of PMCA no PrP^CWD^ amplification in the S96 substrate is detected with neither of the strains. mAb Sha31 1:10,000.

Poor prion propagation by PMCA could be due to low amounts of PrP^CWD^ present in the brain used as a seed^46^. To evaluate the stability of the Wisc-1 and CWD2 strains after passage into transgenic mice, we used brain homogenate from a tg33 mouse challenged with the Wisc-1 strain and brain homogenate from a tgElk mouse^47^ inoculated with the CWD2 strain as seeds for serial propagation in the S96 substrate through 5 rounds of PMCA. Both animals were euthanized at the terminal stage of the disease and accumulated very similar amounts of PrP^CWD^ in the brain (Supplementary Figure 2). After one round of PMCA, tg33 Wisc-1 material amplified up to the 10^−5^ dilution when using the S96 substrate, and the tgElk CWD2 propagated up to the 10^−4^ dilution. When these PMCA products were subjected to a second round of PMCA in the S96 substrate, we observed that PrP^CWD^ amplification was reduced tenfold. After 5 rounds of PMCA, both tg33 Wisc-1 and tgElk CWD2 lost the ability to efficiently propagate in the S96 substrate (Fig. 3).

These results suggest that, although the most common CWD strains found in the wild (Wisc-1 = CWD1; CWD2) can misfold the white-tailed deer S96-PrP^C^, they apparently lack the ability to maintain prion propagation in this substrate, with PrP^CWD^ amplification disappearing after serial passages in the S96-PrP^C^ background. Only the H95^+^ strain was able to serially propagate in the S96-PrP^C^ substrate corroborating the results obtained in vivo^25^.

To evaluate whether the extinction of seeding activity observed with Wisc-1 and CWD2 during serial PMCA in S96-PrP^C^ substrate was related to the loss of affinity for the substrate or due to unstable propagation of alternative conformers competing and blocking each other, we tested for seeding activity in wt-PrP^C^ substrates from tg33 mice. When the products of the 4^th^ PMCA round of Wisc-1 and CWD2 in S96-PrP^C^ substrate were seeded in tg33 wt-PrP^C^ substrate no amplification occurred. Similarly, the products of the 5^th^ PMCA round of tg33-derived Wisc-1 and tgElk-derived CWD2 in the S96 substrate did not amplify or generate protease resistant prion protein in tg33 wt-PrP^C^.

If these strains persisted during serial propagation in the S96-PrP^C^ or if novel conformers emerged due to heterologous prion conversion, it is likely that these amplification products would seed wt-PrP^C^ substrate in subsequent PMCA rounds since it is a more favorable substrate for these strains^24^. We did not recover any PrP^CWD^ seeding activity from the 4^th^ round and 5^th^ round products of Wisc-1 and CWD2 in S96 PrP^C^ following two rounds of PMCA in wt-PrP^C^ substrate (tg33). This indicates that no infectivity was present after serial propagation with the S96-PrP^C^ and that no novel conformers were generated by either Wisc-1 or CWD2 during in vitro conversion of S96-PrP^C^. None of the amplification replicates (n = 3) for the products of the 4^th^ (deer strains) and 5^th^ (mouse strains) rounds of Wisc-1 and CWD2 in S96-PrP^C^ substrate contained seeding activity amplifiable in wt-PrP^C^, suggesting that Wisc-1 and CWD2 strains gradually lost their ability to misfold the S96-PrP^C^ until replication became extinct and no PrP^CWD^ could be detected. These results contrast with previously reported in vivo data that demonstrated that brains from asymptomatic (PK-res negative) tg60 mice challenged with Wisc-1 strain can still infect tg33 mice albeit with very long incubation periods^24^.

## Discussion

Numerous studies in wild and farmed white-tailed deer populations have demonstrated the importance of the prion protein sequence in the susceptibility to CWD^26–30,48^. This evidence was complemented with experimental infections in deer or transgenic mice with PrP^C^ polymorphisms that slow disease progression and modulate the degree of neuropathology^19,23^. One of these polymorphisms, S96-PrP^C^, is relatively common in white-tailed deer populations and has been suggested to be under selection as a response to CWD epizootics^26,27,31,36^. We show that the Serine (S) at codon 96 leads to the collapse of prion conversion for the most common CWD strains (Wisc-1 and CWD2). We propose that the interlocking at the N-terminus between the invading PrP^CWD^ strain and the S96-PrP^C^ controls efficient prion conversion, strain adaptation and disease susceptibility, considering that the Serine residue is in the N-terminus of the white-tailed deer PrP. Only the prion strain with the most unfolding-resistant N-terminal structure (i.e., H95^+^) was serially propagated on S96-PrP^C^^24^. Conversely, the Wisc-1 PrP^CWD^ is less structurally stable^24^. Our experiments suggest that, when encoded in S96-PrP^CWD^ (after round 1), Wisc-1 and CWD2 strains are less functional than H95^+^ at stably misfolding the S96-PrP^C^, impairing proper PrP^C ^− PrP^CWD^ domain alignment, misfolding efficiency and leading to extinction of the colonizing prion conformers.

There is no effective vaccine or preventive treatment against CWD prions. Therefore, strategies to mitigate CWD have been aimed at detecting positive cervids and in some regions reducing their numbers to lower disease prevalence and potentially reduce horizontal transmission between cervids and decrease human exposure. Breeding programs directed at increasing the frequency of *PRNP* alleles associated with resistance to scrapie, implemented by many European countries since 2001^49^, have been very effective for the control of the disease^14^. These programs are based on the elimination of animals expressing the most susceptible genotypes^50^ and gradual replacement with more resistant sheep through the introduction of breeding males of the ARR/ARR genotype.

While the ARR haplotype confers almost complete resistance to scrapie^51,52^, there is no known deer *PRNP* polymorphism that prevents CWD infection^23,31^. The S96 allele is, despite this, a good candidate for the genetic selection of deer in herds at risk of CWD exposure. After the depopulation of a CWD positive white-tailed deer farm in Wisconsin, it was found that 88% of the deer were positive for PrP^CWD^ accumulation in at least one tissue, and the age of the positive animals ranged from fawns to adult deer. From this cohort, only two adult deer had the S96/S96 *PRNP* genotype, with only one of them showing faint PrP^CWD^ accumulation in the retropharyngeal lymph nodes, while the other animal was negative. Further studies have demonstrated that deer expressing the S96 allele in homozygosity not only are less susceptible to CWD infection, but are also found in a significant earlier stage of the disease than deer of the wt genotype^31,33^. These observations and the older age at which positive S96 deer are detected compared to wt/wt deer, indicate the serine at this codon affects prion conversion resulting in slow disease progression.

These conclusions are consistent with the results of experimental infections under controlled conditions in deer and transgenic mice expressing S96 or wt-PrP^C^^21–25,34^. In contrast, previous in vitro replication experiments in which [35^S^]-labeled recombinant wt or S96 PrP^C^ were exposed to different CWD prions showed that both prion proteins are equally susceptible to misfolding^37^. While, in vivo, the conversion of S96-PrP^C^ into PK-res PrP^CWD^ during Wisc-1 infection in tg60 mice was inefficient and resulted in low levels of protease-resistant prion conformers^24^. These novel S96-PrP^Res^ molecules were unable to adapt in vivo and were unstable when subjected to PMCA with S96-PrP^C^ showing altered PrP^Res^ glycotypes^24^. These results indicated that, although the Wisc-1 strain can persistently replicate, the S96-PrP^CWD^ products are less structurally compatible with the S96-PrP^C^ limiting the adaptation in tg60 mice. Independent transmission experiments support this interpretation for other CWD strains, including S96/S96 deer prions, which transmitted with incomplete attack rates when inoculated into tg33 mice expressing wt-PrP^C^ and failed to adapt in tg60 mice^22^.

In this study, we further evaluated the capacity of S96-PrP^C^ to support the in vitro replication of different CWD strains (i.e., white-tailed deer Wisc-1 and H95^+^ strains, elk CWD2 strain and a mule deer (wt/wt) field CWD isolate). We also compared the ability of S96-PrP^C^ and wt-PrP^C^ substrates to support serial prion propagation. After a single round of PMCA, Wisc-1, H95^+^ and CWD2 strains replicated with similar efficiencies in both substrates, similar results to those obtained in other in vitro replication assays^37^. CWD prions from a wild mule deer (wt/wt genotype) were unable to misfold the S96-PrP^C^, which may indicate lower compatibility between this wt/wt mule deer CWD isolate and S96-PrP^C^ compared to Wisc-1, CWD2 and H95^+^ strains perhaps indicating a different strain in mule deer. Contrastingly, after several rounds of PMCA, there was no detectable amplification in the S96-PrP^C^ reactions seeded with Wisc-1 or CWD2 indicating that these CWD prions lost their replication ability when encoded on S96-PrP^CWD^. Serial propagation of these two strains in wt-PrP^C^ substrate resulted in efficient amplification. The loss of replicative capacity in the S96-PrP^C^ observed with these two strains (Wisc-1 and CWD2) but not with the control H95^+^ strain (propagated in parallel and in the same sonicator), is a phenomenon that has not been described during in vitro propagation of prions under standard serial passage conditions.

Previous transmission studies have hypothesized CWD prion strain diversification or differential strain amplification from natural prion strain mixtures following transmission of cervid prions into transgenic mice overexpressing homologous PrP^C^^42^. To evaluate these propositions, we repeated the serial replication experiment with S96-PrP^C^, seeding this time with brain homogenates from transgenic mice that had succumbed to prion disease after inoculation of Wisc-1 and CWD2 from the deer and elk. Each strain was obtained by passage in hosts of homologous PrP^C^ (tg33 and tgElk lines, respectively) that were euthanized with advanced signs of prion disease and contained similar levels of PrP^CWD^ in the brain. The seeding activity in both tg33 and tgElk mice was approximately equivalent as estimated by limiting dilution and a single round of PMCA. Generally, the amount of prions in the input inocula is a major factor influencing the maximum dilution of brain homogenate detectable by PMCA^46^. Serial PMCA amplification allows the detection of highly diluted prion agents with a sensitivity that could exceed that of the mouse bioassay by 10^4^ to 10^5^-fold^40,41,53,54^. Serial rounds of PMCA in principle always allow the generation of large amounts of PrP^Res^ which is accompanied by an increase in western blot detection of the limiting dilutions of the original seed^54^. Here, however, we observed the opposite effect with the S96 substrate. The PMCA efficiency in generating PrP^Res^ decreased with each round for both strains only while serially propagated in S96-PrP^C^. After 5 rounds of serial PMCA, we did not detect generation of S96-PrP^Res^ with either Wisc-1 or CWD2 confirming the results obtained when the inocula was from deer and elk. No emergence of alternate prion strain conformers (e.g., H95^+^) was observed suggesting faithful strain replication upon transfer of the prion strains between the brains of two host species (white-tailed deer and elk into transgenic mice) with the same PrP^C^.

These results confirm that the white-tailed deer Wisc-1 CWD strain, which is favorably propagated in wt-PrP, suffered a gradual loss of replicative capacity when encoded on S96-PrP^CWD^ indicating the residues directly interacting with the S96-PrP^C^ have less affinity than Wisc-1 encoded on wt-PrP^CWD^. These observations made here reconcile the apparently conflicting results previously obtained by exposing the white-tailed deer S96-PrP^C^ to prions from animals of the wt genotype in vitro and in vivo. We must also consider that in vitro systems cannot reproduce all the pathogenic events occurring in a living organism. Animals can degrade a significant amount of the inoculated prions^55,56^ and certain neuroinflammatory mechanisms are neuroprotective in the early stage of prion disease^57^. These phenomena are not present in in vitro assays, making the conversion of PrP^C^ generally easier and/or more efficient.

The Wisc-1 S96-PrP^Res^ and CWD2 S96-PrP^Res^ generated in vitro are unstable and not able to self-propagate when passed serially, leading to the extinction of prion amplification after several passages. This was confirmed by seeding wt-PrP^C^ substrates with material from the last round of Wisc-1 and CWD2 PMCA in S96-PrP^C^. No amplification was obtained in the wt-PrP^C^, (not shown) indicating that these strains did not propagate at a low level in the S96-PrP^C^ substrate.

These results suggest that the genetic selection for animals of the S96 genotype could be a simple and efficient strategy for the control of CWD in deer populations. These results, however, should be interpreted with caution. The introduction of breeding males of the ARR/ARR genotype in sheep has been efficient in controlling scrapie because the ARR haplotype provides a high degree of disease resistance even in heterozygous animals^58,59^. ARR-PrP^C^, together with other prion proteins, can interfere with prion replication in a dominant negative manner, impairing the misfolding of wt-PrP^C^ when co-expressed in the same organism^60^. Heterozygous wt/S96 deer are susceptible to CWD and show a similar PrP^CWD^ distribution to that of wt/wt deer at the terminal stage of the disease^20^, although they present extended incubation periods^19^. We suggest, therefore, that the beneficial effects of this genetic selection strategy on CWD control would begin to be detected after a certain percentage of S96 homozygotes is reached in the deer population.

Another important factor to consider when proposing strategies for the control of CWD is the prion strain to which the animals would be exposed. The H95^+^ strain maintains its replicative ability after multiple rounds in S96-PrP^C^ substrate (Fig. 2). In vivo assays have also confirmed that hosts expressing S96-PrP^C^ are susceptible to the challenge with this CWD strain^24,25^. Genetic selection for the control of a prion disease would not necessarily protect the animal against all the strains present. For example, sheep expressing the ARR allele are strongly resistant to classical scrapie, but susceptible to atypical scrapie^61–63^. Deer selected for the expression of the S96 allele would still be susceptible to the H95^+^ strain^19,24,25,36^. It is possible, therefore, that selecting for deer of the S96-PrP^C^ may initially have beneficial effects on CWD control, but this also could lead to the selection of other prion strains, such as H95^+^, which would be able to propagate between deer of this genotype. The H95^+^ strain has yet to be described in wild deer populations since this strain emerged following experimental transmission in deer expressing H95 PrP^C^^24,25^. In addition, the H95-*PRNP* allele has been described in low frequency in several CWD enzootic regions of North America^26,27,31^. An increase in the allele frequencies of protective *PRNP* alleles in response to chronic CWD exposure can in turn result in better conditions for other strains to emerge^25,35,36^.

In conclusion, S96 allele plays an important role in CWD resistance, possibly because most common prion strains (Wisc-1 and CWD2) cannot propagate stably misfolded S96-PrP^C^ in vitro*.* Our data demonstrates that most common CWD prions do not maintain their replicative capacity when serially passaged in S96-PrP^C^ substrates in vitro, which is consistent with in vivo data for the Wisc-1 strain^24^. This suggests that even if a deer of the S96 genotype could be infected with these strains, transmission to other animals of the same genotype may not occur. Therefore, selective breeding is a potential strategy for controlling the spread of CWD. However, these strategies could favor emergence of less abundant strains with ability to propagate in more resistant genotypes.

## Material and methods

### CWD isolates

Wisc-1 and H95^+^ isolates consisted of 10% brain homogenates (wt/vol in water) obtained from the brain of terminally ill, orally infected white-tailed deer (*Odocoileus virginianus*) expressing the wild-type (Q95/G96) and H95/S96 PrP^C^, respectively^19,25^. These isolates have been thoroughly characterized in transgenic mice, wild type mice and hamsters^25,43^. There is no evidence demonstrating that Wisc-1 and the previously described CWD1 strain are different strains since they show identical glycosylation patterns and similar incubation periods and neuropathological features in transgenic mice^25,42^.

CWD2 isolate was prepared from a pool of brains from three CWD positive elk (*Cervus canadensis*) of the MM132 genotype^44^ and was generously provided by Dr. Catherine Graham. This elk pool was characterized as CWD2 strain in previous studies using transgenic mice expressing deer PrP^C^^42^.

Mule deer (*Odocoileus hemionus*) CWD (MD CWD isolate) was obtained from the obex region of a hunter-harvested deer of the wild type *PRNP* genotype (animal ID 102565) and provided by Dr. Margo Pybus (Alberta Environment and Parks). This animal was culled in the 2017 Alberta hunting season and tested for CWD by Alberta Environment and Parks. PrP^CWD^, as detected by western blot, was abundant in the brain material from this deer, showing a glycosylation profile indistinguishable from Wisc-1 strain.

Tg33 Wisc-1 isolate consisted of a 10% brain homogenate (wt/vol in water) from a tg33 transgenic mouse that had developed terminal prion disease after the inoculation of deer Wisc-1 CWD (369 days post-inoculation)^25^. TgElk CWD2 seed was a 10% (wt/vol) brain homogenate from a transgenic mouse overexpressing elk PrP^C^^47^ euthanized at the terminal stage of prion disease (110 days post-inoculation) after the challenge with the CWD2 elk pool.

Experimental procedures in mice were conducted in accordance with the Canadian Council on Animal Care Guidelines, Policies with approval from Animal Care and Use Committee: Health Sciences at the University of Alberta and ARRIVE guidelines.

### Protein misfolding cyclic amplification of CWD isolates

PMCA was performed as described previously^45^. PMCA was performed using brain homogenates from uninfected tg33 and tg60 mice as substrates. Tg33 mice express the wild-type PrP^C^ from white-tailed deer (G96) at levels ~ 1 × than those found in the deer brain. Tg60 mice express the white-tailed deer S96 PrP^C^, at levels ~ 0.7 × than those detected in the tg33 transgenic line. Both transgenic lines have the same strain background^21,22^. After euthanasia by isoflurane inhalation, tg33 and tg60 mice were perfused using ice-cold PBS + 5 mM EDTA. Perfused brains were immediately frozen at − 80 ºC. Brain substrate (10% w/v brain homogenate) was prepared using a tissue grinder, and the brains were homogenized in PMCA conversion buffer (PBS + 150 mM NaCl, 1% Triton X-100 + 0.5 M EDTA, 1 × Protease Inhibitor Cocktail). Aliquots of 90 µl were prepared with brain homogenates from tg60 mice and stored at − 80 ºC. Since tg60 mice express 30% less PrP^C^ than tg33 mice, tg33 substrates were diluted with a perfused brain homogenate from *Prnp*^*0/0*^ mice, obtaining substrates with an equivalent PrP^C^ amount.

For the titration of the CWD agents, CWD seeds were diluted from 10^2^ to 10^7^ -fold in conversion buffer. Then, 10 µl of each dilution were used to seed 90 µl of tg33 or tg60 substrate. Three PTFE beads (3/32″; McMaster-Carr) were added to each PMCA reaction to increase the efficiency of prion amplification^39^. Seeded substrates were placed on the plate holder of a S-4000 Misonix sonicator (QSonica, Newtown, CT, USA) and subjected to one round of PMCA consisting of incubation cycles of 15 min at 37 ºC without shaking, followed by sonication pulses of 30 s at 60% power. After a round of 24 h of PMCA, samples were analyzed for PrP^CWD^ amplification.

Analysis of serial amplification of Wisc-1, CWD2, H95^+^ and MD deer CWD isolates in wt or S96-PrP^C^ was performed by diluting the CWD seeds 10^3^-fold into 10% normal tg33 or tg60 brain homogenates. Each of these reactions was seeded in triplicate and carried out in the same sonicator, using identical PMCA conditions. Reactions were subjected to PMCA for 24 h. After this first round, 10 µl of the PMCA products were diluted into 90 µl of fresh substrate and sonicated again, repeating this process through 4 rounds of PMCA. Tg33 Wisc-1 and tgElk CWD2 seeds were serially diluted (10^−3^ to 10^−7^) in the tg60 substrate and propagated for 5 rounds of PMCA. Both serial PMCA experiments were performed twice obtaining the same results.

### Biochemical analysis of PMCA products

PMCA amplified samples were protease digested using 50 µg/ml PK during 1 h at 37 ºC with constant agitation (700 rpm). Digestion was terminated by the addition of 2 × Laemmli sample buffer (150 mM Tris–HCl, pH 6.8, 0.5% bromophenol blue, 25% glycerol, 5% [wt/vol] SDS, 12.5% ß-mercaptoethanol) and boiling at 100 ºC for 10 min. Samples were analyzed by western blot. Immunodetection of PrP^CWD^ was performed with mouse monoclonal antibody Sha31 (1:10,000; Cayman Chemical) and alkaline phosphatase-conjugated goat anti-mouse secondary antibody (1:10,000; Promega). Blots were developed using the AttoPhos AP Fluorescent Substrate System (Promega).

## Supplementary Information

Supplementary Information.

## Data Availability

Data available within the article and its supplementary materials.
