# Supplementary material for: White-tailed deer S96 prion protein does not support stable in vitro propagation of most common CWD strains
Source: Sci Rep. 2021 May 27;11:11193. doi: 10.1038/s41598-021-90606-8 (PMC8160261; doi:10.1038/s41598-021-90606-8)

**Supplementary material**

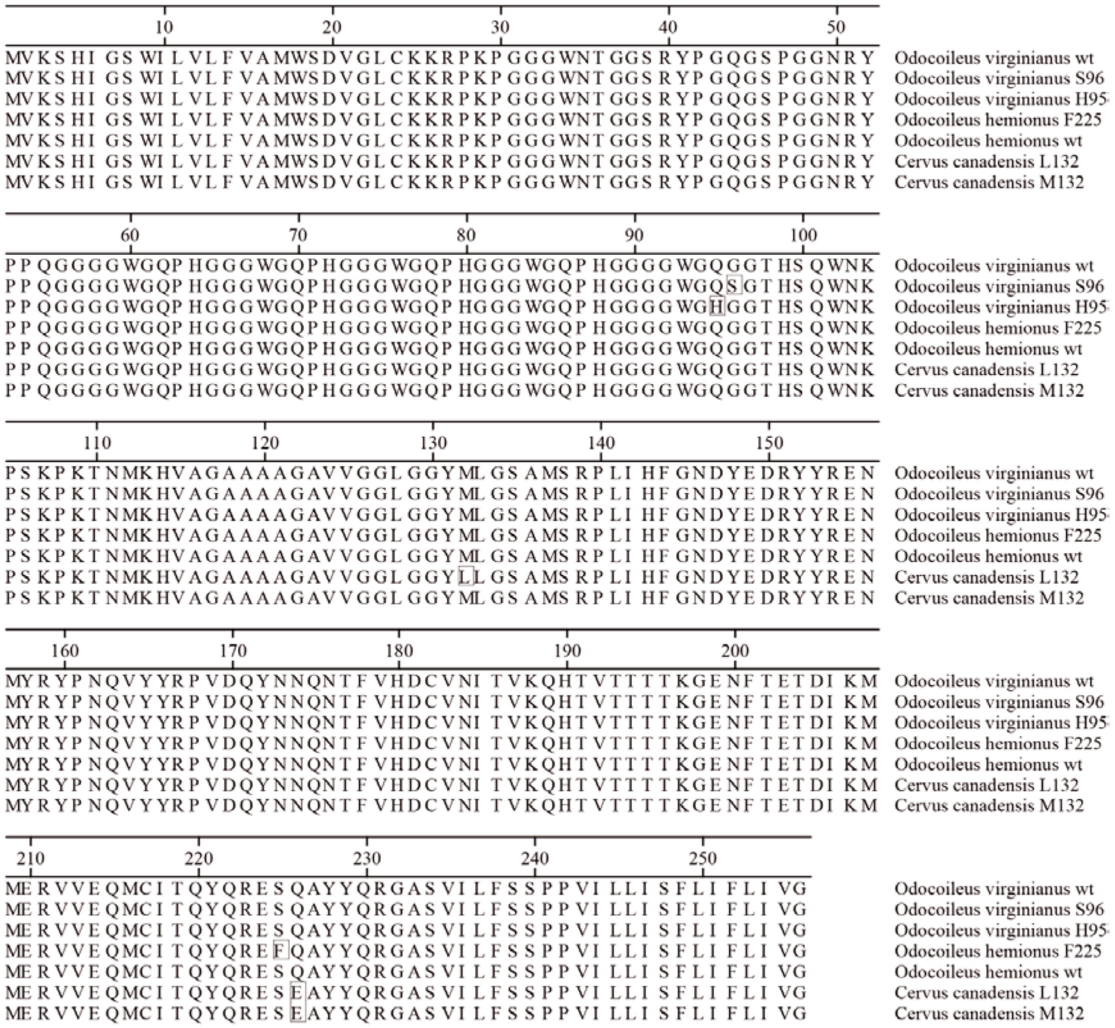


**Supplementary figure 1: Sequence alignment of PrP$^{C}$ primary structure and occuring polymorphisms within and between commonly affected cervid species.** Boxes delimit species-specific amino acid polymorphisms.

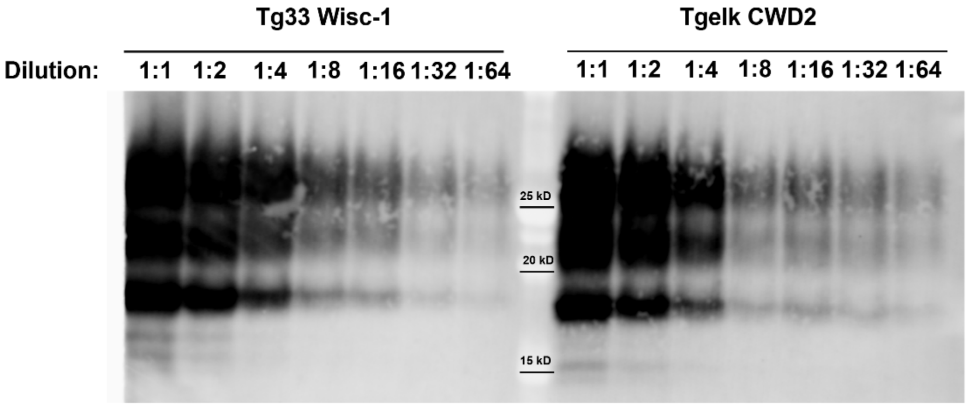


**Supplementary figure 2: Detection of $PrP^{CWD}$ in brain samples from a tg33 mouse challenged with Wisc-1 CWD and a tgElk mouse challenged with CWD2 strain.** 10% brain homogenates from these mice were serially diluted and then digested with 50 µg/ml of Proteinase-K (PK). Digested samples were analyzed by western blot using the Sha31 monoclonal antibody (1:10000). Both animals accumulated similar amounts of $PrP^{CWD}$ in their brains.

Full-length western blots corresponding to the main figures in the text are provided on the following pages.

**Main figure 1 Wisc-1 (left):**

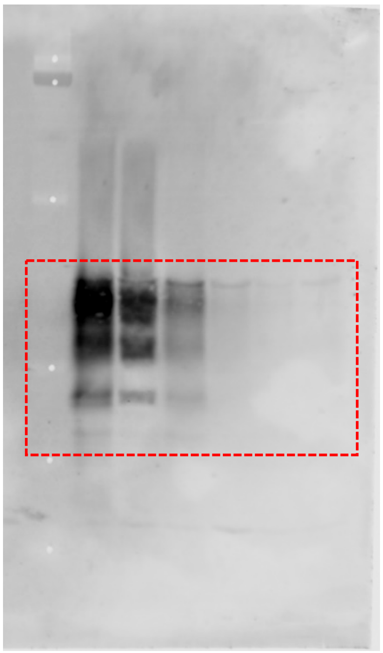

**Main Figure 1 Wisc-1 (right):**

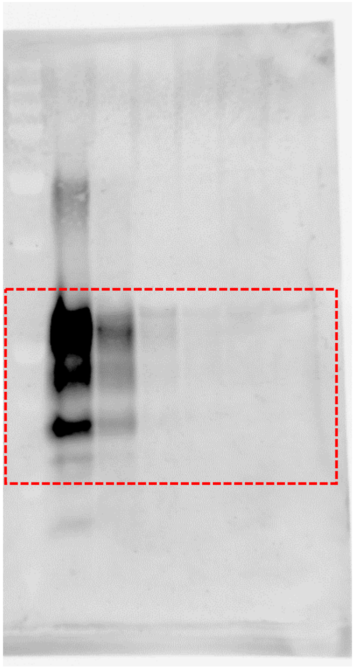

**Main figure 1 H95+ (left and right):**

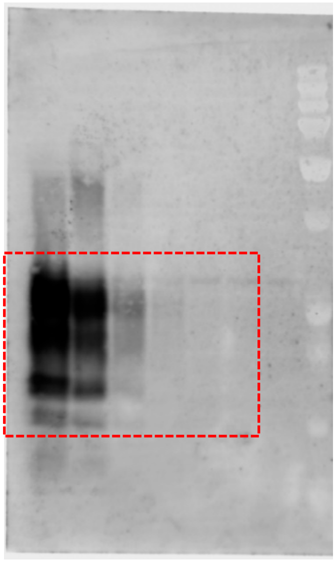

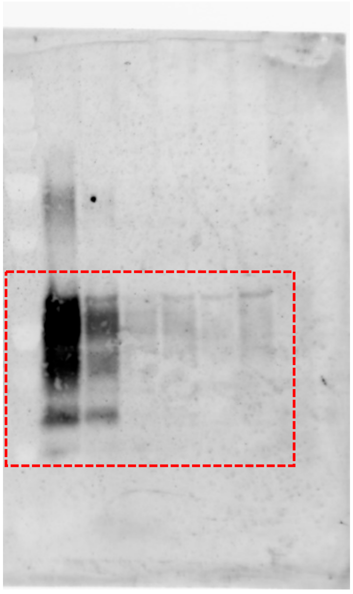

## Main figure 1 CWD2 (left and right)

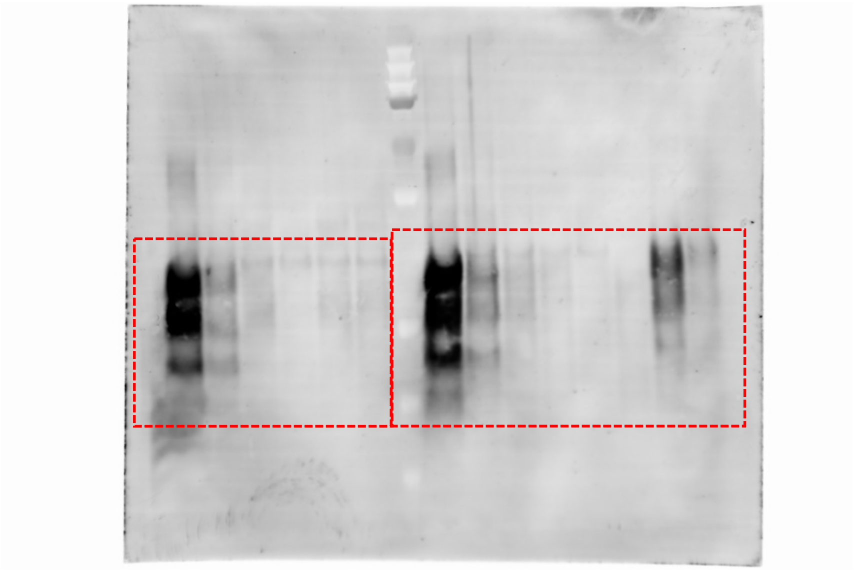

## Main figure 1MD (left and right):

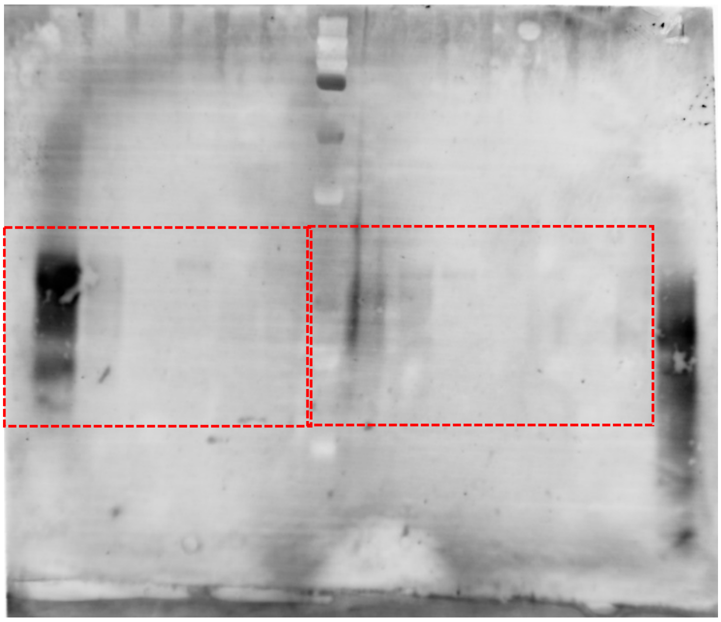

**Main figure 2 Wisc-1:**

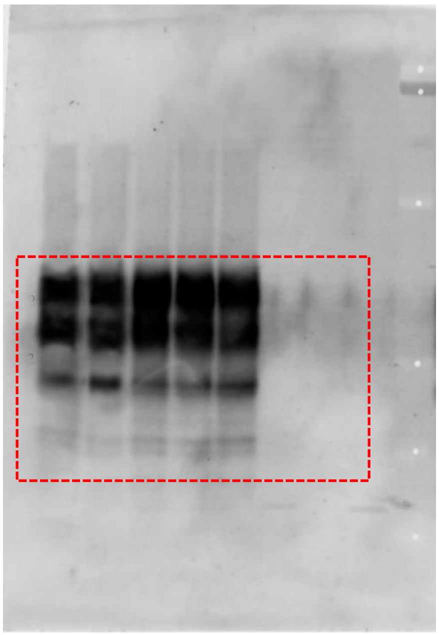

**Main figure 2 CWD2:**

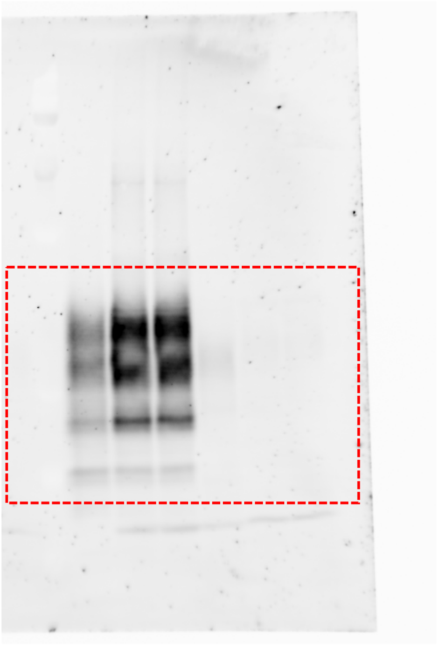

**Main figure 2 H95+ and MD:**

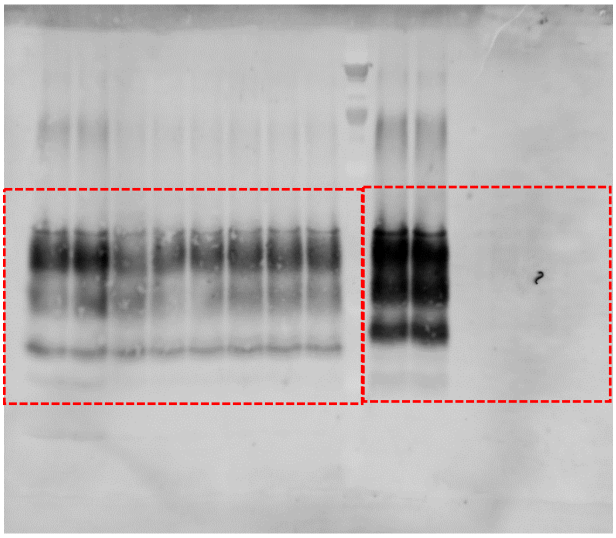

## Main figure 3 top panels:

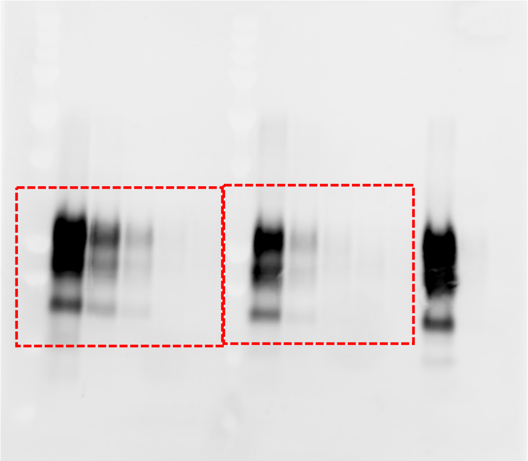

## Main figure 3 mid panels:

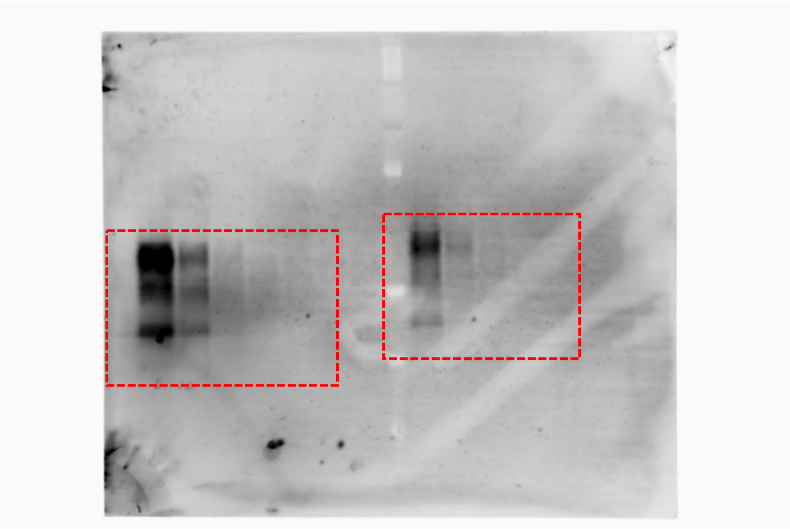

## Main figure 3 low panels:

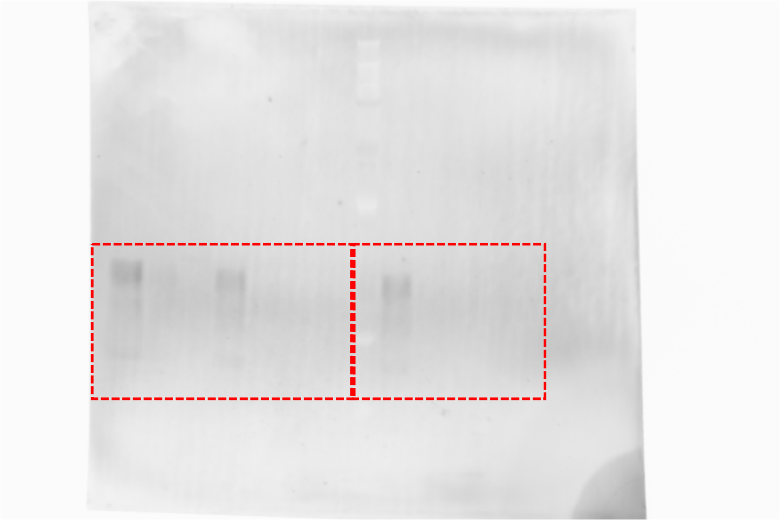

Supplement: Supplementary file 1 — Supplementary Information. [file 41598_2021_90606_MOESM1_ESM.pdf]
